# Bulge-Derived Epithelial Cells Isolated from Human Hair Follicles Using Enzymatic Digestion or Explants Result in Comparable Tissue-Engineered Skin

**DOI:** 10.3390/ijms26051852

**Published:** 2025-02-21

**Authors:** Bettina Cattier, Rina Guignard, Israël Martel, Christian Martel, Carolyne Simard-Bisson, Danielle Larouche, Béatrice Guiraud, Sandrine Bessou-Touya, Lucie Germain

**Affiliations:** 1 Centre de Recherche en Organogénèse Expérimentale de l’Université Laval/LOEX, Quebec City, QC G1J 5B3, Canada; bettina.cattier@crchudequebec.ulaval.ca (B.C.); rina.guignard@crchudequebec.ulaval.ca (R.G.); israel.martel@crchudequebec.ulaval.ca (I.M.); christian.martel@crchudequebec.ulaval.ca (C.M.); carolyne.simard-bisson@crchudequebec.ulaval.ca (C.S.-B.); danielle.larouche@crchudequebec.ulaval.ca (D.L.); 2 Department of Surgery, Faculty of Medicine, Université Laval, Quebec City, QC G1V 0A6, Canada; 3 CHU de Québec-Université Laval Research Centre, Quebec City, QC G1J 5B3, Canada; 4 R&D Center, Pierre Fabre Dermo-Cosmétique, 31100 Toulouse, France; beatrice.guiraud@pierre-fabre.com (B.G.); sandrine.bessou.touya@pierre-fabre.com (S.B.-T.)

**Keywords:** hair follicle, stem cells, regenerative medicine, tissue-engineered skin, cell culture technique, primary cell culture

## Abstract

Hair follicle stem cells, located in the bulge region of the outer root sheath, are multipotent epithelial stem cells capable of differentiating into epidermal, sebaceous gland, and hair shaft cells. Efficient culturing of these cells is crucial for advancements in dermatology, regenerative medicine, and skin model development. This investigation aimed to develop a protocol for isolating enriched bulge-derived epithelial cells from scalp specimens to produce tissue-engineered substitutes. The epithelium, including hair follicles, was separated from the dermis using thermolysin, followed by microdissection of the bulge region. Epithelial stem cells were isolated using enzymatic dissociation to create a single-cell suspension and compared with the direct explant culture and a benchmark method which isolates cells from the epidermis and pilosebaceous units. After 8 days of culture, the enzymatic digestion of microdissected bulges yielded 5.3 times more epithelial cells compared to explant cultures and proliferated faster than the benchmark method. Cells cultured from all methods exhibited comparable morphology and growth rates. The fully stratified epidermis of tissue-engineered skin was similar, indicating comparable differentiation potential. This enzymatic digestion method improved early-stage cell recovery and expansion while maintaining keratinocyte functionality, offering an efficient hair bulge cell-extraction technique for tissue engineering and regenerative medicine applications.

## 1. Introduction

**Figure 1:**
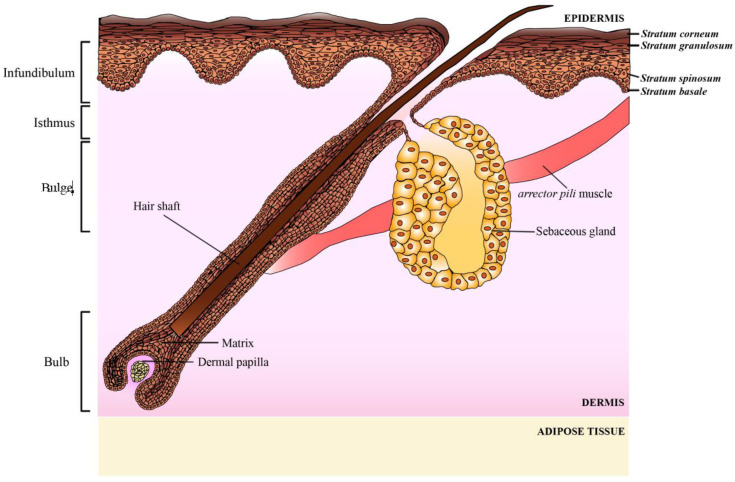
Schematic representation of human skin and the pilosebaceous unit. The multilayered structure of the epidermis, dermis, and adipose tissue, along with the hair follicle and associated sebaceous gland, are illustrated. The epidermis is stratified into the basal, spinous, granular, and cornified layers. The hair follicle is divided into distinct anatomical regions: the infundibulum, isthmus, bulge, and bulb. The bulge region, known to harbor epithelial stem cells, is located at the insertion point of the arrector pili muscle. The sebaceous gland, connected to the hair follicle, plays a role in sebum production. The dermal papilla, situated at the base of the follicle within the bulb, is essential for hair follicle regeneration.

The human skin epidermis has a unique capacity for continuous renewal and repair, driven by its resident stem cell populations. These cells are distributed across different compartments of the skin, including the basal layer of the interfollicular epidermis, hair follicles, and sebaceous glands [1]. These progenitors reside in a specialized microenvironment called the niche, which is essential for preserving their stemness and regenerative potential [2,3]. Among these compartments, hair follicles are dynamic, self-renewing structures that undergo cyclic phases of growth and degeneration (anagen, catagen, telogen) [4,5]. Hair follicle stem cells are localized in the outer root sheath (ORS) at the level of the bulge, surrounding the hair shaft, between the sebaceous gland insertion point and the arrector pili muscle attachment [6,7] (Figure 1). Several markers have been identified in these cells isolated from humans, including keratin 19, keratin 15, and CD200 [8]. The bulge region serves as a reservoir of multipotent epithelial stem cells with the ability to differentiate into various cell types, including epidermal cells, sebaceous gland cells, and hair shaft cells [7].

The development of methods for effectively culturing skin epithelial cells has been a significant step in research for both fundamental and clinical applications. Their expansion in vitro has led to important advances in many areas, particularly in dermatology, regenerative medicine, and biotechnology [9,10,11,12]. The pioneering work of Rheinwald and Green on the Cultured Epithelial Autografts (CEAs) [9] led to the first successful clinical application of cultured epithelial cells [13]. Over time, skin substitute technology has evolved to integrate both dermal and epidermal components, improving models for studying epithelial differentiation and skin regeneration [11,14]. In addition, hair follicles have gained attention as an abundant and promising source of autologous stem cells, harboring both multipotent and pluripotent populations [15]. Bulge-derived epithelial cells offer significant potential, as they are accessible in adults and present high differentiation and regenerative capacities [6,16]. Beyond hair regeneration to treat alopecia, recent preclinical and clinical studies have highlighted their capacity to support tissue regeneration [15]. Preclinical studies in rats show that hair follicle stem cell injections accelerate wound healing and vascularization, while clinical applications like EpiDex^®^ demonstrate efficacy in treating chronic ulcers [17,18]. More broadly, the ability of mouse bulge cells to differentiate into osteoblasts, cardiomyocytes, and neurons under specific culture conditions highlights their potential in regenerative medicine [19,20,21,22,23]. Optimizing hair follicle stem cells isolation and culturing is crucial to fully harness their therapeutic potential.

Hair follicles can be collected through plucking [24,25,26] or from scalp tissues obtained after cosmetic surgery with the patient’s informed consent [27,28,29,30]. While minimally invasive, plucking may leave a portion of the bulge attached to the dermis, resulting in incomplete retrieval of the stem cell-rich compartment and providing a modest yield in terms of cell number per hair [31,32]. Otherwise, enzymatic digestion of full-thickness scalp specimens using thermolysin effectively separates the dermis from the epidermis by targeting the dermal–epidermal junction [33,34]. This process facilitates the easy detachment of the epidermis and pilosebaceous units prior to epithelial cell dissociation. Therefore, thermolysin allows for the preservation of stem cells while minimizing fibroblast contamination in the cultures, offering a higher quality and quantity of cells [33,34]. Access to the various skin epithelial stem cell compartments then becomes possible. Following digestion, hair follicles can be dissected under a microscope, focusing on isolating the stem cell-rich bulge region [30]. The area just below the midpoint of the follicle contains a concentration of colony-forming cells, highlighting its richness in cells with regenerative potential [35]. The mid-section of the hair follicle can be manually microdissected based on its distinct morphology. However, without additional purification steps, such as flow cytometry or magnetic bead sorting, a portion of transit-amplifying cells or differentiated keratinocytes may be included in the isolated population [6]. Although microdissection does not yield a pure population of stem cells [6], it remains effective for isolating specific regions of the hair follicle and enriching the bulge-derived stem cell population.

Given the importance of culturing skin epithelial stem cells to study their differentiation potential into epidermis, hair, and sebaceous glands, the objective of this study was to develop a protocol allowing for the efficient dissociation of hair bulge epithelial cells from human scalp specimens to produce tissue-engineered skin. We hypothesized that the enzymatic digestion of microdissected bulges using trypsin instead of culturing them as explants would provide a better yield in terms of cells that can be expanded in culture. This protocol proposed the use of thermolysin to initiate the dermal–epidermal separation, followed by hair follicle isolation and then microdissection to segment the bulge region with or without further digestion. The yields in terms of epithelial cell number and the colony-forming efficiency over passages were compared. Their functionality was assessed by producing tissue-engineered skin substitutes by the self-assembly method to evaluate the epidermal differentiation [36,37].

## 2. Results

**Figure 2:**
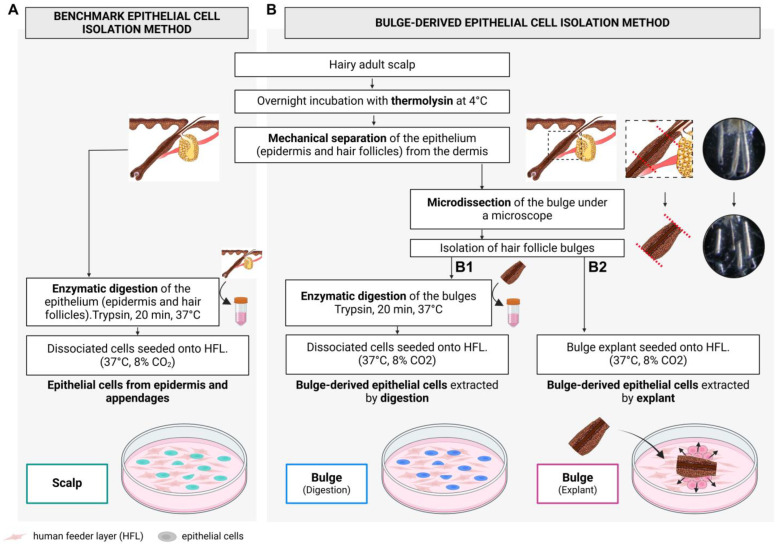
Flowchart of methodological approaches used to obtain epithelial cell populations from human scalp tissue. Scalp tissue was cut into small pieces and incubated overnight with thermolysin. After separating the epithelium from the dermis, epithelial cells from the epidermis and hair follicles were either extracted using the two-step thermolysin and trypsin keratinocyte-isolation procedure that served as the benchmark method (**A**) or microdissected to enrich for bulge-derived epithelial cells (**B**). After microdissection, bulge-derived cells were isolated using two different protocols; (**B1**) bulges were digested with trypsin to obtain a single-cell suspension, or (**B2**) bulges were cultivated as explants. For all conditions, cells were cultured on a human feeder layer (HFL) in keratinocyte medium. Created in BioRender. De koninck, H. (2025) https://BioRender.com/c48t805 (accessed on 29 December 2024).

This study compared the quality of primary cultures of epithelial cells extracted from scalp specimens using three methods. The experimental design is illustrated in Figure 2. After dermal–epidermal separation using thermolysin, hair follicles were isolated and segmented at the level of the bulge. Microdissected bulges were either digested with trypsin to dissociate cells prior to seeding (referred to as digested bulge) or cultured as explants (referred to as explanted bulge). Cell cultures obtained from these two methods were compared to those obtained using the two-step thermolysin and trypsin keratinocyte-isolation procedure that served as the benchmark method [24]. With this third method, scalp epithelial cells were isolated from the epidermis and from hair follicles (referred to as scalp).

**Figure 3:**
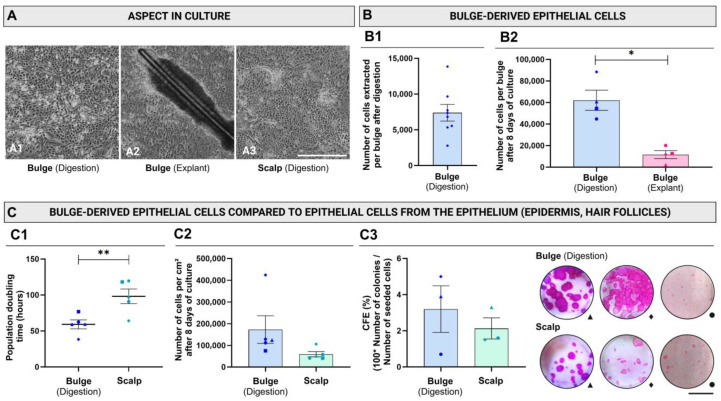
Effect of the isolation method on the number of cells and colony-forming efficiency after the extraction and in primary culture. (**A**) Phase-contrast microscopy of epithelial cells derived from trypsin-digested bulges (**A1**), explanted bulges (**A2**), or trypsin-digested cells extracted from scalp (epidermis and hair follicles) (**A3**) after 6 days of culture. Pictures show cells from the same donor. (**B**) Number of bulge-derived epithelial cells obtained according to the isolation method used at the extraction step (**B1**) and extrapolated for 8 days of culture (**B2**). (**C**) Comparison of cells isolated by digestion from the bulge or from the scalp. Population doubling time of epithelial cells derived from digested bulge and from scalp after primary culture (**C1**) and number of epithelial cells per cm^2^ extrapolated for 8 days of culture (**C2**). Colony-forming efficiency (CFE) after the primary culture. (**C3**). Scale bar = 1 cm (**A**,**C3**). Each point represents a different donor (N). Detailed information about the donors, identified by their corresponding symbols, is provided in the table in the Section 4.1. The quantification of CFE (%) was performed in at least three technical replicates (n). Data are presented as mean ± standard error of the mean (SEM). Statistical analysis: bilateral *t*-test (**B2**,**C1**,**C3**) or Wilcoxon test (**C2**) for paired samples. (*) *p*-value < 0.05, (**) *p*-value < 0.01.

The impact of the extraction method on the morphological aspect of the cells by phase contrast microscopy was first evaluated. Cells derived from digested bulges (Figure 3(A1)), explanted bulges (Figure 3(A2)), and scalps (Figure 3(A3)) exhibited similar characteristics in primary cultures. They formed colonies showing cells with polygonal morphology and a high nuclear-to-cytoplasm ratio.

The advantage of enzymatic digestion over the explant method was the possibility of counting the cells after extraction and seeding them at a given cell density. An average of 7400 (standard error of the mean (SEM) = 2300) viable cells per bulge were obtained after digestion (Figure 3(B1)). In contrast, for explant cultures, the initial cell density has to be estimated, since it could not be measured because the bulge-derived cells migrated outward from the bulge tissue; they were not dissociated (Figure 3(A2)). The time necessary for cells to reach 80–90% confluency was not synchronized across the tested conditions. Therefore, to better compare the cell yield of the primary culture for cells obtained using the different methods, we calculated the number of cells generated per bulge after 8 days of culture using doubling time (see Section 4). After 8 days in culture, the number of viable epithelial cells per bulge was 62,200 (standard error of the mean (SEM) = 13,200) for digested bulge, compared to 11,700 (SEM = 4800) for explanted bulge (Figure 3(B2)).

The population doubling time was compared between digested bulges and scalp-derived cells. Cells extracted from the bulges exhibited a 1.7 times lower population doubling time (98.2 h (SEM = 16.6)) compared to scalp-derived cells (59.3 h (SEM = 8.5)), indicating significantly faster proliferation (Figure 3(C1)). Using the population doubling time, the cell yield after 8 days was extrapolated (see Section 4) and compared. The mean yield of cells from digested bulges was 2.9-fold higher than the one from the scalp (172,900 (SEM = 100,500) cells/cm^2^ compared to 59,400 (SEM = 18,700) cells/cm^2^, respectively—*p*-value = 0.0625) (Figure 3(C2)).

Colony-forming efficiency (CFE) reflects the proliferative capacity of the cells [38]. No statistically significant difference was obtained between digested bulge-derived cells and those from the scalp (Figure 3(C3)). It was not possible to measure CFE from explanted bulge, as it requires cell suspension.

**Figure 4:**
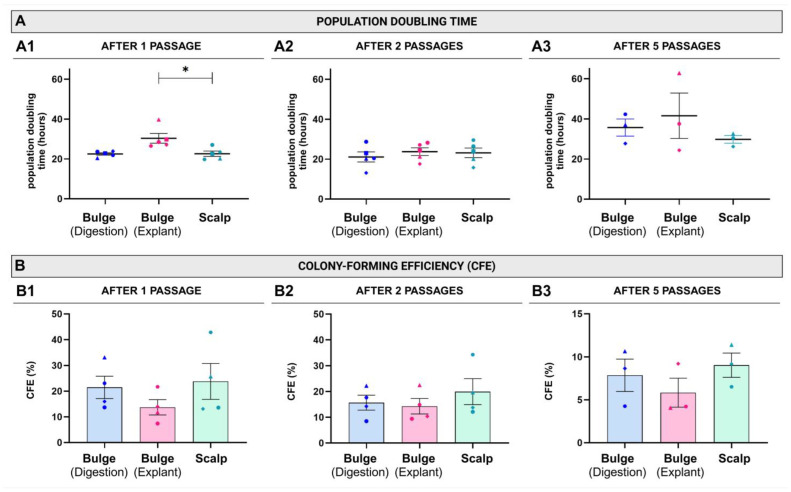
Effect of the extraction method on the cell population doubling time and colony-forming efficiency over cell passages. (**A**) The population doubling time of epithelial cells derived from digested bulges, explanted bulges, or from scalps (epidermis and hair follicles) after 1 (**A1**), 2 (**A2**), and 5 (**A3**) passages. (**B**) Comparative colony-forming efficiency (CFE) of cells extracted with the 3 methods after 1 (**B1**), 2 (**B2**) and 5 (**B3**) passages. Each point represents a different donor (N). Detailed information about the donors, identified by their corresponding symbols, is provided in the table in Section 4.1. Data are presented as mean ± standard error of the mean (SEM). Statistical analysis: Friedman test with Dunn’s multiple comparisons (**A1**) and one-way ANOVA with Tuckey’s multiple comparisons (**A2**,**A3**,**B1**–**B3**). * *p*-value < 0.05.

The impact of the tested extraction methods on the growth rate and clonogenic capacity of the cells over successive passages was evaluated by determining the population doubling time and colony-forming efficiency (CFE). After the first passage (P1), the population doubling time was significantly higher in explanted bulge-derived cells compared to those derived from scalps (Figure 4(A1)). When compared to digested bulges, the population doubling time was longer for explanted bulges for a given donor, but no statistically significant difference was detected between the means of aggregated data (Figure 4(A1), *p* = 0.17). No statistically significant difference was observed between the population doubling time of digested bulge- and scalp-derived cells (Figure 4(A1)). For subsequent subcultures (passages 2 and 5), the population doubling time and the CFE were similar for the three groups (*p* > 0.3 for all comparisons) (Figure 4(A2,A3),B, and Supplementary Figure S1).

**Figure 5:**
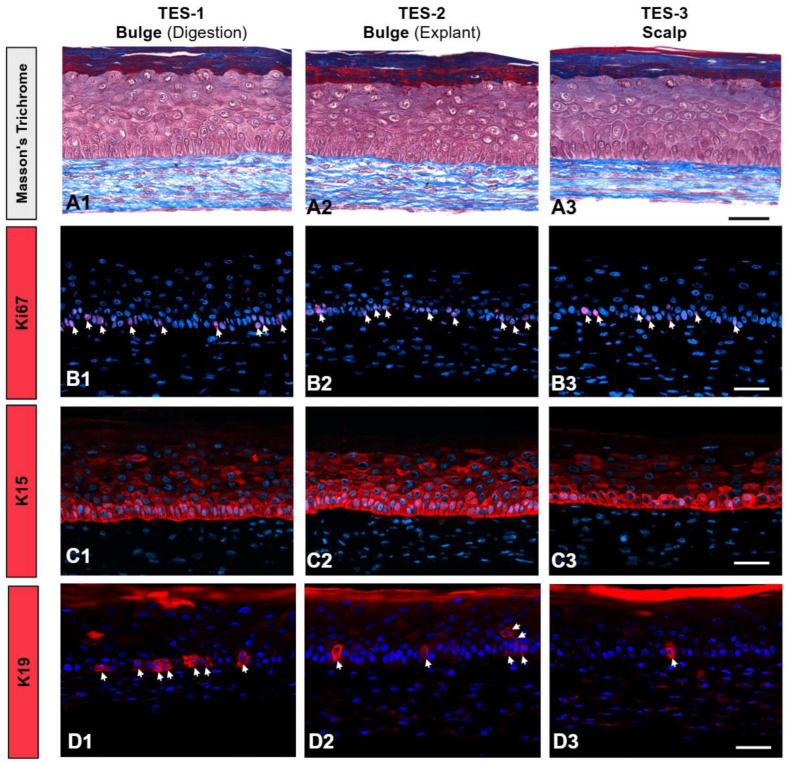
Effect of the extraction method on the production of tissue-engineered skin substitutes (TESs). TESs were produced with scalp fibroblasts and keratinocytes obtained from digested bulges (TES-1, **A1**,**B1**,**C1**,**D1**), explanted bulges (TES-2, **A2**,**B2**,**C2**,**D2**), or from scalp (epidermis, hair follicles) (TES-3, **A3**,**B3**,**C3**,**D3**). (**A1**–**A3**) Masson’s Trichrome staining (colors the cells in pink and extracellular matrix in blue). (**B1**–**B3**) Immunostaining of Ki67 (red, white arrows). (**C1**–**C3**) Immunostaining of keratin 15 (K15, red). (**D1**–**D3**) Immunostaining of keratin 19 (red, white arrows). Pictures are representative of TESs made with cells from two different donors (see the table in Section 4.1—⬣,●). Nuclei are stained with Hoechst (blue). Scale bars = 50 µm.

The impact of the extraction methods on epithelial cell functionality was assessed by evaluating their epidermal differentiation potential through the generation of tissue-engineered skin substitutes (TESs) using the self-assembly approach developed in our laboratory [39]. Histological analysis showed that TESs produced with each of the three epithelial cell populations displayed the typical architecture of a stratified epidermis with the four expected layers, including the *stratum corneum* (Figure 5(A1–A3)), indicating a fully differentiated epidermis. The proliferation marker Ki-67 [40] was detected in the nucleus of a proportion of keratinocytes within the basal layer of all TESs (Figure 5(B1–B3)), see white arrows), indicating active keratinocyte proliferation in the epidermis. Similarly, keratin 15 (K15), a progenitor cell-associated marker [41,42], was comparably expressed across all TESs (Figure 5(C1–C3)). The stem cell marker keratin 19 (K19) [43] was expressed in a small subset of basal keratinocytes in all TESs (Figure 5(D1–D3), see white arrows), indicating the persistence of stem cells in the reconstructed epidermis. Interestingly, TES generated from bulge-derived cells showed a trend toward higher K19 expression.

**Figure 6:**
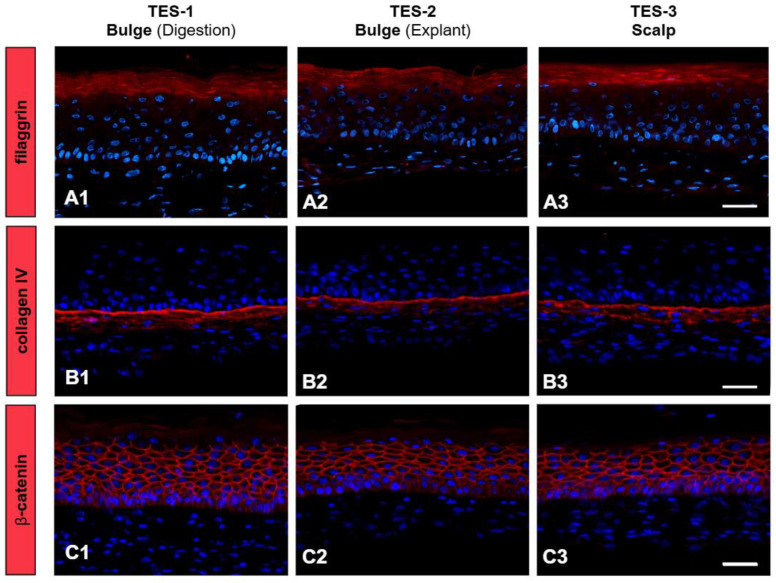
Effect of the extraction method on the dermal–epidermal junction and epidermal differentiation (TES). TESs were produced with scalp fibroblasts and keratinocytes obtained from digested bulges (TES-1, **A1**,**B1**,**C1**), explanted bulges (TES-2, **A2**,**B2**,**C2**), or from scalp (epidermis, hair follicles) (TES-3, **A3**,**B3**,**C3**). (**A1**–**A3**) Immunostaining of filaggrin (red). (**B1**–**B3**) Immunostaining of collagen IV (red). (**C1**–**C3**) Immunostaining of β-catenin (red). Pictures are representative of TESs made with cells from two different donors (see the table in Section 4.1—⬣,●). Nuclei are stained with Hoechst (blue). Scale bars = 50 µm.

The integrity of the dermal–epidermal junction and epidermal differentiation within the TES was evaluated (Figure 6). Filaggrin, a key protein of the cornified envelope [44,45], was detected in the upper layers of the epidermis in all TES conditions (Figure 6(A1–A3)), indicating that the cell-isolation method did not impair epidermal maturation. A continuous type IV collagen deposition was detected at the dermal–epidermal junction in all TESs (Figure 6(B1–B3)), confirming the basement membrane integrity. β -catenin was used to validate the distribution of adherent junctions at the cell membrane of keratinocytes [46]. β-catenin was detected around keratinocytes in all conditions (Figure 6(C1–C3)). Together, these findings confirm that TESs generated from all extraction methods exhibit key markers of epidermal differentiation, proliferation, and adhesion, supporting their potential for regenerative applications.

## 3. Discussion

Our results demonstrate that single epithelial cells isolated by the digestion of microdissected bulges result in skin substitutes of high quality, comparable to those produced with epithelial cells cultured from scalp (epidermis and hair follicles) or from microdissected bulge explants. Our protocol for digesting hair bulge yielded 5.3 times more epithelial cells at the end of the primary culture compared to cultured explants. Interestingly, cells extracted from the bulge proliferated 1.7 times faster than epithelial cells digested from the entire scalp epithelium (epidermis and hair follicles) during the primary culture. These findings align with previous research highlighting the higher in vitro proliferative potential of hair follicle stem cells, particularly those from the upper follicle region compared to interfollicular epidermal cells [47]. Notably, similar phenomena have been observed in rodent hair follicles, where the upper bulge exhibits strong proliferative capabilities [48,49]. In vivo, the upper bulge plays a critical role in maintaining the entire pilosebaceous unit, as recent studies have demonstrated its involvement in sustaining hair follicle and sebaceous gland functionality through β-catenin regulation [50,51,52]. The key features of skin epithelial cells assessed, including morphology, growth rate, colony-forming efficiency, and the ability to produce a fully stratified epidermis, revealed the quality and functionality of our cell cultures. Regardless of the extraction method, all cultures exhibited cells with comparable polygonal morphology, with a high proportion of cells showing a high nucleus-to-cytoplasm ratio, indicative of undifferentiated epithelial cells [38,53].

The proliferation potential remained unaffected by the extraction method, as evidenced by the consistent growth rate during cell passages. The difference in population doublings between extraction methods was most pronounced during primary culture (P0) but diminished over subsequent passages, with proliferation rates stabilizing by passage five regardless of the method used (Supplementary Figure S1). By allowing cell numbering and the control of initial seeding densities, the proposed hair bulge-digestion method is better suited for experiments conducted on primary cultures. However, the microdissection step remains a limitation of this method since it requires additional time (approximately 1.5 h to isolate 50 bulges) and qualified personnel compared to the benchmark method.

The entire hair follicle or the isolated bulge region can be processed through enzymatic dissociation to create a single-cell suspension or cultured directly as an explant. Explant cultures of plucked hair follicles [25] have been effective for producing an epidermal equivalent [12]. Other studies have utilized enzymatic digestion to isolate bulge-derived cells [27,28,54]. The slightly higher yield we obtained could be due to the use of thermolysin instead of dispase to induce the dermal–epithelial separation. Thermolysin was shown to result in more colony-forming efficiency (CFE) than trypsin and dispase [55].

In our study, we deliberately chose to employ a microdissection approach to preserve the cellular diversity within the bulge, which contains a mix of stem and differentiated cells [56]. By preserving this heterogeneity, our approach ensures a broader representation of the bulge’s epithelium regenerative potential. Other protocols using trypsin digestion included an additional step to enrich stem cell populations using magnetic beads or flow cytometry to selectively enrich CD200+ cells, a marker that has been associated with bulge-derived epithelial cells [8,27,57]. By using magnetic beads to selectively enrich CD200+ and to deplete other markers (CD24, CD34, CD71, CD146), Ohyama et al. have reported a 3% CFE (approximatively 150 colonies/T25 flasks, seeded with 5000 cells) [27]. In comparison, our protocol provided a higher colony-forming efficiency, with an average of 4% CFE in P0, reaching up to 10% in passage 1 (P1) and subsequent passages. In a second study, the average doubling time reported for the enriched CD200+ cells was 38.3 days, compared to 2.5 days in the present study [28]. In addition to the absence of a purification step, the higher proliferation potential may also be attributed to the use of a human feeder layer for cell cultivation. Therefore, our protocol allows obtaining epithelial cell cultures of high quality while achieving superior growth metrics.

While the explant culture method is effective, it has several limitations. This method relies on the migration of cells out of the explant, followed by their growth as a monolayer, which forms a front that moves across the culture dish, rather than the growth of colonies from evenly distributed single cells. The process of sorting cells from the explant likely takes time, and it is possible that not all cells migrate out. This results in fewer cells proliferating on the plastic compared to single cells directly seeded onto the Petri dish after enzymatic digestion. In the latter case, cells can readily attach and divide after seeding. This could explain why we observed a lower total cell number at the end of the primary culture (P0). We noted that the explant-derived cells were harder to detach after the primary culture; two sequential trypsin incubations were required to maximize the cell yield, a step unnecessary with enzymatic digestion. These findings align with those from Orazizadeh et al. [58], who demonstrated that enzymatic tissue digestion enhances initial cell recovery compared to the explant of foreskin keratinocytes, making it a more efficient method for protocols requiring higher initial cell densities at low passage. The cell yield after the primary culture for bulge cultured from explants had to be estimated using the initial cell recovery of digested bulges; this extrapolation may have led to an overestimation of the cell yield for the explant method.

One limitation of this study was that our skin samples were obtained predominantly from women over 40 years old, which may limit the generalization of the results to the broader population. Recent study on plucked follicles has shown that age and sex jointly influence cell expansion, particularly in individuals over 40 years old [24]. This study has reported that women’s cells exhibited lower outgrowth success than men’s cells. In our study, by performing matched comparisons based on the cell donor, our experimental design accounted for inter-individual variability that might affect the conclusions regarding the difference in efficiency between tested methods. In the future, more diverse donor populations, balanced in terms of age and sex, could be compared to determine whether our bulge-digestion method provides the same advantages compared to bulge explant cultures regardless of age and sex.

The quality of the tissue-engineered skin substitutes (TESs) produced from epithelial cells obtained with the three isolation methods combined with fibroblasts aligned with previous observations showing that plucked hairs could produce a stratified epidermis [12]. In our study, TESs generated from in vitro expanded bulge-derived epithelial cells—whether isolated by explant culture or enzymatic digestion—demonstrated proper epidermal differentiation. They formed a mature dermal–epidermal junction, which is essential for maintaining skin substitute integrity. The presence of proliferative cells in the basal layer, their differentiation as they migrated upwards, and the production of filaggrin in the upper layers successfully mimicked the physiological process occurring in the native epidermis. These outcomes were comparable to that of our benchmark method, which is currently used to produce skin substitutes in an ongoing clinical trial for the treatment of severe burn patients [11]. In our TESs, β-catenin was expressed at cell membranes consistent with its role in cell–cell adhesion [59].

Moving forward, obtaining an enriched population of bulge cells has various applications. Recent findings have further expanded our understanding of hair follicle stem cells by identifying a multipotent population located in the upper region of the hair follicle near the sebaceous gland, referred to as hair follicle-associated pluripotent (HAP) stem cells. These cells have been shown to possess the ability to differentiate into various cell types beyond the follicle, including nerve cells, endothelial cells, and adipocytes [60]. Their potential involvement in sebaceous gland differentiation suggests they could play a crucial role in skin appendage regeneration [51]. In regenerative medicine, their multipotency could be harnessed to improve the functional aspect of bioengineered skin grafts by enhancing the formation of appendages such as hair follicles and sebaceous glands—critical elements for skin homeostasis and function. Enriched bulge cells, which contain a high concentration of multipotent stem cells, hold potential for the generation of skin appendages within reconstructed skin models.

Although the ability of human bulge stem cells to induce hair follicle formation outside their native environment remains a challenge [61], advancing our understanding of hair follicle stem cell culture is essential to uncover strategies for preserving their functionality in vitro. In 2018, Abaci et al. achieved a milestone by successfully regenerating human hair follicles in vitro [62]. This innovation marked a significant shift, as previous studies had only demonstrated hair follicle regeneration in vivo [16,63,64,65,66]. A model like that of Abaci, or the self-assembled skin substitute developed by our team that sustains hair follicle stem cell preservation [67], could be useful tools to study how biochemical and mechanical cues guide epithelial stem cell fate. Advancing our understanding of hair follicle stem cell culture is crucial to preserving their functionality in vitro. By incorporating human bulge-derived stem cells into these models, we may gain deeper insights into the mechanisms governing hair follicle and sebaceous gland formation, ultimately paving the way for more effective therapeutic strategies in skin tissue engineering.

## 4. Materials and Methods

### 4.1. Tissue Collection

Scalp samples were collected from leftover skin from facelift surgeries. Exclusion criteria included pregnancy or breastfeeding, recent use of hair care products, inflammatory skin conditions (e.g., psoriasis, seborrheic dermatitis), alopecia, recent radiotherapy, chemotherapy, or previous scalp surgery. Participants who had received treatments for alopecia, dermatological conditions, or long-term use of anti-inflammatory drugs, corticosteroids, or antibiotics during the four weeks prior to surgery were excluded. The cell populations used in this study are detailed in Table 1.

### 4.2. Skin Cell Isolation

#### 4.2.1. Total Epithelium: Interfollicular Epidermis, Hair Follicles, and Sebaceous Glands

Primary epithelial cells were isolated as previously described [34,68]. Briefly, skin samples were digested for 13 to 16 h at 4 °C in thermolysin (500 µg/mL, Sigma-Aldrich, Oakville, ON, Canada or 3000 U/mL, Roche, Laval, QC, Canada) dissolved in a HEPES buffer. Skin pieces were transferred into a droplet of phosphate-buffered saline (PBS) supplemented with MgCl_2_/CaCl_2_ and dissected under a binocular microscope to separate the epithelium (epidermis and hair follicles) from the dermis. After mechanical separation, the epidermis was further digested in trypsin (0.05%, Gibco, Waltham, MA, USA)–EDTA (0.01%, Avantor, Radnor, PA, USA) for 25 min at 37 °C. The cells were then centrifuged at 300× *g* for 10 min and counted using a Coulter Z2 (Beckman, Mississauga, ON, Canada) with the viability evaluated using trypan blue (Sigma-Aldrich). For the primary culture (P0), epithelial cells were seeded at a density ranging from 10,000 to 40,000 cells/cm^2^ on a human feeder layer (HFL) of irradiated dermal fibroblasts, seeded at a density of 8000 cells/cm^2^, and prepared as described [69]. Cells were incubated in keratinocyte medium composed of Dulbecco–Vogt Modified Eagle’s (Gibco) and Ham’s F12 (Gibco) (ratio 3:1) Medium supplemented with 24.3 µg/mL adenine (Sigma-Aldrich), 5 µg/mL insulin (Millipore Sigma, Oakville, ON, Canada), 0.4 µg/mL hydrocortisone (Galenova, Saint-Hyacinthe, QC, Canada), 0.212 µg/mL isoproterenol hydrochloride (Sigma-Aldrich), 5% Hyclone Fetal Clone II bovine serum (Cytiva, Marlborough, MA USA), 10 ng/mL human epidermal growth factor (R&D Systems, Minneapolis, MN, USA), 100 U/mL penicillin (Sigma-Aldrich), and 25 μg/mL gentamycin (MP Biomedicals, Solon, OH, USA) at 37 °C, 8% CO₂. The medium was changed every 2–3 days until the culture reached 80–95% confluence.

#### 4.2.2. Bulge-Derived Cell Isolation: Microdissection

The initial digestion using thermolysin was performed as described above. Using a scalpel (#22 blades, Aspen Surgical, Caledonia, MI, USA), the bulge region was cut above the hair bulb and below the sebaceous gland (mid-part of the hair follicle). The bulge was either collected by capillary action or gently grasped with forceps and transferred to keratinocyte medium.

#### 4.2.3. Bulge-Derived Cells Isolation: Enzymatic Digestion

Collected bulges were centrifuged (300× *g*, 10 min), resuspended in 5 mL trypsin (0.05%, Gibco)–EDTA (0.01%, Avantor), gently vortexed, and incubated for 20 min at 37 °C. During this incubation, bulges were vortexed every 5 min. Digestion was stopped with 5 mL of keratinocyte medium, and the contents were mixed. The cells were then centrifuged and counted using a Coulter Z2 (Beckman). The percentage of dead cells was evaluated with trypan blue. The pellet was resuspended in 1 mL of keratinocyte medium and seeded in T25 flasks or in 6-well plate onto HFL. Cells were incubated at 37 °C, 8% CO₂. The medium was changed every 2–3 days until the culture reached 85% confluence.

#### 4.2.4. Bulge-Derived Cells Isolation: Explant Approach

The collected bulges were centrifuged, resuspended in 1 mL of keratinocyte medium, and briefly vortexed. The tube content was then rapidly distributed onto a 6-well plate containing HFL, from which the medium had been removed. To facilitate the settling of explants, they were cultured in 0.5 mL for 2–3 h. Once the explants had adhered, 0.5 mL of fresh keratinocyte medium was added. The cultures were incubated at 37 °C with 8% CO₂, and the medium was replaced every 2–3 days for up to 10 days.

### 4.3. Subcultures

When the cells reached 80–95% confluence or after maximum 10 days, trypsin (0.05%, Gibco)–EDTA (0.01%, Avantor) was used to detach the cells. Cells were seeded at a density of 6500 cells/cm^2^ onto HFL. Cells were subcultured up to five passages (P).

### 4.4. Skin Substitute Production

Tissue-engineered skin substitutes (TESs) were produced using epithelial cells isolated by three different methods after amplification. The tissue-engineered skin substitutes (TESs) were produced following the protocol by Larouche et al., 2016 [39], adapted for 3.5 cm diameter TESs. The same TES production protocol was used for all groups, ensuring that any observed differences are solely due to variations in the epithelial cell-extraction process. Fibroblasts were seeded in Petri dishes at a density of 8000 cells/cm^2^ and cultured in fibroblast medium (Dulbecco’s Modified Eagle Medium (DMEM, Gibco) supplemented with 10% Avantor Seradigm FB Essence serum (Avantor), 100 U/mL penicillin (Sigma-Aldrich), and 25 μg/mL gentamycin (MP Biomedicals)), supplemented with 50 μg/mL ascorbic acid (Sigma). The medium was refreshed every 2 to 3 days. Cells were cultured until the formation of a manipulable extracellular matrix-rich tissue sheet (approximately 25 days). Dissociated epithelial cells from all groups were seeded at a density of 200,000 cells/cm^2^ onto a fibroblast-derived tissue sheet. The tissues were then cultured for 4 days in keratinocyte medium supplemented with 50 µg/mL ascorbic acid (Sigma). Following this culture period, each tissue containing fibroblasts and epithelial cells was stacked onto 2 fibroblast-derived tissue sheets. The resulting skin substitutes were then positioned at the air–liquid interface and cultured for an additional 10 days in keratinocyte medium without epidermal growth factor and supplemented with 50 μg/mL ascorbic acid.

### 4.5. Histology and Immunofluorescence

For histological analysis, skin samples were fixed in 3.7% formaldehyde (ACP Chemicals, Saint-Léonard, QC, Canada) and embedded in paraffin before sectioning with a microtome into 5 µm slices. Sections were stained with Masson’s trichrome to examine tissue morphology.

For immunostaining, skin samples were embedded in Tissue-Tek OCT Compound (Sakura, Finetek, Torrance, CA, USA). Cryosections (5 µm) were then fixed and permeabilized using cold acetone at −20 °C for 10 min. Following fixation, tissue sections were immunostained with primary antibodies specific to keratin 15 (K15, 1:1000, Sigma, #A57944), Ki67 (1:400, BD Biosciences, Mississauga, On, Canada, #556003), mouse monoclonal (IgG2a) anti-human keratin 19, clone A53-B/A2 (gift from Dr. Uwe Karsten, Institute of Biological Sciences, University of Rostock, Rostock, Germany), filaggrin (1:400, Abcam, Waltham, MA, USA, #ab81468), beta-catenin (1:100, Santa Cruz Biotechnology, Dallas, TX, USA, #SC-7963), and collagen IV (1:500, Institut Pasteur, Lyon, France) for 45 min at room temperature. After primary antibody incubation, tissue sections were rinsed and treated with fluorochrome-conjugated secondary antibodies—anti-rabbit IgG(H + L) conjugated to Alexa Fluor 594 (1:500 or 1:1000, #A11012, ThermoFisher, Mississauga, ON, Canada) and anti-mouse IgG(H + L) conjugated to Alexa Fluor 594 (1:1400, Life technologies, #A11005)—and Hoechst 33258 (Sigma) for 45 min under the same conditions. To assess antibody specificity, only the secondary antibody was used on a control sample. Images were captured using an Axio Imager.Z2 microscope (Zeiss, Oberkochen, Germany).

### 4.6. Population Doubling Time and Cell-Yield Evaluation After the First Passage (P0)

When possible (digested bulge and scalps), cells were seeded at a known initial density and cultured for a defined period. After this period, the total number of cells was counted as described above, and the doubling time was calculated using Equation (1).

Population doubling time (hours) = (culture time (hours)) × ln(2)/ln(final cell number/initial cell number) (1)

To calculate the population doubling time of cells cultured from explanted bulges, we first had to estimate the initial cell number. For each donor, we took the number of cells per bulge isolated after digestion (Figure 2(B1)) multiplied by the number of explants seeded.

The Equation (2) was used to estimate the cell yield per bulge after 8 days of culture.

Cell yield per bulge after 8 days = Number of cells obtained per bulge after digestion × exp(8 days × 24 hours × ln(2)/population doubling time (hours))(2)

Since the time to reach 80–90% confluency was not the same for each cell population, we used the population doubling time to calculate the cell yield per cm^2^ after 8 days.

Cell yield per cm^2^ after 8 days = (1,000,000 cells/75 cm^2^) × exp(8 days × 24 hours × ln(2)/population doubling time (hours)). (3)

### 4.7. Colony-Forming Efficiency (CFE)

The colony-forming efficiency was evaluated at passages 0, 1, 2 and 5 (P0, P1, P2, P5). For each condition, epithelial cells were seeded at the same density (P0: 134 cells per cm^2^; P1, P2, P5: 20–60 cells per cm^2^) and cultured for the same number of days. Keratinocyte medium was changed at day 5 and 7, and cultures were fixed on day 11 (P0) or day 9 (P1, P2, P5) with 3.7% formaldehyde (ACP Chemicals) overnight at room temperature. Colonies were then stained with 1.5 mL of 1% Rhodamine for 15 min at room temperature. Colonies were rinsed with distilled water, dried, and manually counted. Equation (4) was applied to calculate the colony-forming efficiency (CFE).

CFE (%) = (Number of colonies/Initial number of keratinocytes seeded) × 100(4)

### 4.8. Statistical Analysis

Microsoft Excel was used for the primary analysis of raw data, while GraphPad Prism 8 (San Diego, CA, USA) was employed for statistical analysis and graph generation. The normality of the data was assessed using the Shapiro–Wilk test. For comparisons between two conditions, a paired *t*-test was used if the data were normally distributed. For comparisons between three conditions, a one-way ANOVA followed by Tukey’s multiple comparisons test was applied if the data were normally distributed. If the data were not normally distributed, a Wilcoxon test or a Friedman test with Dunn’s multiple comparisons test was used.

## 5. Conclusions

In conclusion, our study highlighted the critical influence of the extraction method on the quality of primary epithelial cell cultures from tissue specimens. The enzymatic digestion of hair bulge sections demonstrated a clear advantage over explant culture and cultures of epithelial cells extracted from the epidermis and hair follicles, offering a higher number of viable cells and a higher proliferative capacity at low passages, resulting in a higher expansion potential. The enzymatic digestion method facilitates efficient experimental setups while preserving the functional characteristics of the extracted bulge epithelial cells, ensuring their suitability for downstream applications. These findings emphasize the practical benefits of enzymatic digestion for protocols prioritizing early-stage cell expansion while maintaining long-term culture viability, establishing it as a valuable method for applications in tissue engineering and regenerative medicine therapies. We concluded that bulge-derived epithelial cells hold promising regenerative properties, offering significant potential for the development of advanced 2D and 3D skin models and innovative therapeutic applications. Isolating these cells and understanding their behavior in vitro will help refine protocols to enhance their regenerative potential and clinical applicability.

## Figures and Tables

**Table 1:** Epithelial cell populations and donor information.

Sex	Age (y.o. ^1^)	Color Code Digested or Explanted Bulge, Scalp ^2^
Male	64	▲ ▲ ▲
Female	61	● ● ●
Female	55	⬣ ⬣ ⬣
Female	68	♦ ♦ ♦
Female	61	■ ■ ■
Female	60	● Digestion
Female	63	
Female	71	
	Mean = 63 (S.D. ^3^ = 5)	

^1^ y.o.: years old; ^2^ Cell-extraction methods (see Section 4.2.1, Section 4.2.2, Section 4.2.3 and Section 4.2.4); ^3^ S.D.: standard deviation.

## Data Availability

The data presented in this study are available upon request to the corresponding author.

## Supplementary Materials

The following are available online at https://www.mdpi.com/article/10.3390/ijms26051852/s1.

## References

[1] Kretzschmar K., Watt F.M. (2014). Markers of Epidermal Stem Cell Subpopulations in Adult Mammalian Skin. Cold Spring Harb. Perspect. Med..

[2] Gonzales K.A.U., Fuchs E. (2017). Skin and Its Regenerative Powers: An Alliance between Stem Cells and Their Niche. Dev. Cell.

[3] Blanpain C., Lowry W.E., Geoghegan A., Polak L., Fuchs E. (2004). Self-Renewal, Multipotency, and the Existence of Two Cell Populations within an Epithelial Stem Cell Niche. Cell.

[4] Schneider M.R., Schmidt-Ullrich R., Paus R. (2009). The Hair Follicle as a Dynamic Miniorgan. Curr. Biol..

[5] Stenn K.S., Paus R. (2001). Controls of Hair Follicle Cycling. Physiol. Rev..

[6] Ohyama M. (2007). Hair Follicle Bulge: A Fascinating Reservoir of Epithelial Stem Cells. J. Dermatol. Sci..

[7] Cotsarelis G., Sun T.-T., Lavker R.M. (1990). Label-Retaining Cells Reside in the Bulge Area of Pilosebaceous Unit: Implications for Follicular Stem Cells, Hair Cycle, and Skin Carcinogenesis. Cell.

[8] Kloepper J.E., Tiede S., Brinckmann J., Reinhardt D.P., Meyer W., Faessler R., Paus R. (2008). Immunophenotyping of the Human Bulge Region: The Quest to Define Useful in Situ Markers for Human Epithelial Hair Follicle Stem Cells and Their Niche. Exp. Dermatol..

[9] Rheinwald J.G., Green H. (1975). Serial Cultivation of Strains of Human Epidermal Keratinocytes: The Formation of Keratinizing Colonies from Single Cells. Cell.

[10] Wüstner L.S., Klingenstein M., Frey K.G., Nikbin M.R., Milazzo A., Kleger A., Liebau S., Klingenstein S. (2022). Generating IPSCs with a High-Efficient, Non-Invasive Method—An Improved Way to Cultivate Keratinocytes from Plucked Hair for Reprogramming. Cells.

[11] Germain L., Larouche D., Nedelec B., Perreault I., Duranceau L., Bortoluzzi P., Cloutier C.B., Genest H., Caouette-Laberge L., Dumas A. (2018). Autologous Bilayered Self-Assembled Skin Substitutes (Sasss) as Permanent Grafts: A Case Series of 14 Severely Burned Patients Indicating Clinical Effectiveness. Eur. Cell Mater..

[12] Guiraud B., Hernandez-Pigeon H., Ceruti I., Mas S., Palvadeau Y., Saint-Martory C., Castex-Rizzi N., Duplan H., Bessou-Touya S. (2014). Characterization of a Human Epidermis Model Reconstructed from Hair Follicle Keratinocytes and Comparison with Two Commercially Models and Native Skin. Int. J. Cosmet. Sci..

[13] O’Connor N.E., Mulliken J.B., Banks-Schlegel S., Kehinde O., Green H. (1981). Grafting of Burns with Cultured Epithelium Prepared from Autologous Epidermal Cell. Lancet.

[14] Cortez Ghio S., Larouche D., Doucet E.J., Germain L. (2021). The Role of Cultured Autologous Bilayered Skin Substitutes as Epithelial Stem Cell Niches after Grafting: A Systematic Review of Clinical Studies. Burn. Open.

[15] Peterson A., Nair L.S. (2021). Hair Follicle Stem Cells for Tissue Regeneration. Tissue Eng. Part B Rev..

[16] Ji S., Zhu Z., Sun X., Fu X. (2021). Functional Hair Follicle Regeneration: An Updated Review. Signal Transduct. Target. Ther..

[17] Cheng X., Yu Z., Song Y., Zhang Y., Du J., Su Y., Ma X. (2020). Hair Follicle Bulge-Derived Stem Cells Promote Tissue Regeneration during Skin Expansion. Biomed. Pharmacother..

[18] Goedkoop R.J., Hunziker T. (2002). EPIDEX, A NOVEL TREATMENT FOR RECALCITRANT CHRONIC LEG ULCERS. ASAIO J..

[19] Mignone J.L., Roig-Lopez J.L., Fedtsova N., Schones D.E., Manganas L.N., Maletic-Savatic M., Keyes W.M., Mills A.A., Gleiberman A., Zhang M.Q. (2007). Neural Potential of a Stem Cell Population in the Hair Follicle. Cell Cycle.

[20] Yashiro M., Mii S., Aki R., Hamada Y., Arakawa N., Kawahara K., Hoffman R.M., Amoh Y. (2015). From Hair to Heart: Nestin-Expressing Hairfollicle- Associated Pluripotent (HAP) Stem Cells Differentiate to Beating Cardiac Muscle Cells. Cell Cycle.

[21] Urano-Morisawa E., Takami M., Suzawa T., Matsumoto A., Osumi N., Baba K., Kamijo R. (2017). Induction of Osteoblastic Differentiation of Neural Crest-Derived Stem Cells from Hair Follicles. PLoS ONE.

[22] Amoh Y., Li L., Katsuoka K., Penman S., Hoffman R.M. (2005). Multipotent Nestin-Positive, Keratin-Negative Hair-Follicle Bulge Stem Cells Can Form Neurons. Proc. Natl. Acad. Sci. USA.

[23] Amoh Y., Li L., Campillo R., Kawahara K., Katsuoka K., Penman S., Hoffman R.M. (2005). Implanted Hair Follicle Stem Cells Form Schwann Cells That Support Repair of Severed Peripheral Nerves. Proc. Natl. Acad. Sci. USA.

[24] Fatehi A., Sadat M., Fayyad M., Tang J., Han D., Rogers I.M., Taylor D. (2024). Efficient Generation of Pancreatic Progenitor Cells from Induced Pluripotent Stem Cells Derived from a Non-Invasive and Accessible Tissue Source—The Plucked Hair Follicle. Cells.

[25] Limat A., Noser F.K. (1986). Serial Cultivation of Single Keratinocytes from the Outer Root Sheath of Human Scalp Hair Follicles. J. Investig. Dermatol..

[26] Gho C.G., Braun J.E.F., Tilli C.M.L.J., Neumann H.A.M., Ramaekers F.C.S. (2004). Human Follicular Stem Cells: Their Presence in Plucked Hair and Follicular Cell Culture. Br. J. Dermatol..

[27] Ohyama M., Terunuma A., Tock C.L., Radonovich M.F., Pise-Masison C.A., Hopping S.B., Brady J.N., Udey M.C., Vogel J.C. (2006). Characterization and Isolation of Stem Cell-Enriched Human Hair Follicle Bulge Cells. J. Clin. Investig..

[28] Oh J.H., Mohebi P., Farkas D.L., Tajbakhsh J. (2011). Towards Expansion of Human Hair Follicle Stem Cells in Vitro. Cell Prolif..

[29] Guo Z., Draheim K., Lyle S. (2011). Isolation and Culture of Adult Epithelial Stem Cells from Human Skin. J. Vis. Exp..

[30] Molina B.J., Finol H.J. (2020). Isolation, Cultivation, and Morphological Characteristics of Hair Follicle Adult Stem Cells in the Bulge Region in Mouse and Human. Microsc. Res..

[31] Li H., Masieri F.F., Schneider M., Bartella A., Gaus S., Hahnel S., Zimmerer R., Sack U., Maksimovic-Ivanic D., Mijatovic S. (2021). The Middle Part of the Plucked Hair Follicle Outer Root Sheath Is Identified as an Area Rich in Lineage-Specific Stem Cell Markers. Biomolecules.

[32] Kumar A., Gupta S., Mohanty S., Bhargava B., Airan B. (2013). Stem Cell Niche Is Partially Lost during Follicular Plucking: A Preliminary Pilot Study. Int. J. Trichology.

[33] Walzer C., Benathan M., Frenk E. (1989). Thermolysin Treatment: A New Method for Dermo-Epidermal Separation. J. Investig. Dermatol..

[34] Germain L., Rouabhia M., Guignard R., Carrier L., Bouvard V., Auger F.A. (1993). Improvement of Human Keratinocyte Isolation and Culture Using Thermolysin. Burns.

[35] Rochat A., Kobayashi K., Barrandon Y. (1994). Location of Stem Cells of Human Hair Follicles by Clonal Analysis. Cell.

[36] Lavoie A., Fugère C., Fradette J., Larouche D., Paquet C., Beauparlant A., Gauvin R., Têtu F.A., Roy A., Bouchard M. (2011). Considerations in the Choice of a Skin Donor Site for Harvesting Keratinocytes Containing a High Proportion of Stem Cells for Culture in Vitro. Burns.

[37] Michel M., L’heureux N., Pouliot R., Xu W., Auger F.A., Germain L. (1999). Characterization of a New Tissue-Engineered Human Skin Equivalent with Hair. In Vitro Cell. Dev. Biol.-Anim..

[38] Barrandon Y., Green H. (1987). Three Clonal Types of Keratinocyte with Different Capacities for Multiplication. Proc. Natl. Acad. Sci. USA.

[39] Larouche D., Cantin-Warren L., Desgagné M., Guignard R., Martel I., Ayoub A., Lavoie A., Gauvin R., Auger F.A., Moulin V.J. (2016). Improved Methods to Produce Tissue-Engineered Skin Substitutes Suitable for the Permanent Closure of Full-Thickness Skin Injuries. Biores. Open Access.

[40] Gerdes J., Schwab U., Lemke H., Stein H. (1983). Production of a Mouse Monoclonal Antibody Reactive with a Human Nuclear Antigen Associated with Cell Proliferation. Int. J. Cancer.

[41] Liu Y., Lyle S., Yang Z., Cotsarelis G. (2003). Keratin 15 Promoter Targets Putative Epithelial Stem Cells in the Hair Follicle Bulge. J. Investig. Dermatol..

[42] Lyle S., Christofidou-Solomidou M., Liu Y., Elder D.E., Albelda S., Cotsarelis G. (1998). The C8/144B Monoclonal Antibody Recognizes Cytokeratin 15 and Defines the Location of Human Hair Follicle Stem Cells. J. Cell Sci..

[43] Michel M., Török N., Godbout M.J., Lussier M., Gaudreau P., Royal A., Germain L. (1996). Keratin 19 as a Biochemical Marker of Skin Stem Cells in Vivo and in Vitro: Keratin 19 Expressing Cells Are Differentially Localized in Function of Anatomic Sites, and Their Number Varies with Donor Age and Culture Stage. J. Cell Sci..

[44] Kalinin A.E., Kajava A.V., Steinert P.M. (2002). Epithelial Barrier Function: Assembly and Structural Features of the Cornified Cell Envelope. Bioessays.

[45] Steinert P.M., Cantieri J.S., Teller D.C., Lonsdale-Eccles J.D., Dale B.A. (1981). Characterization of a Class of Cationic Proteins That Specifically Interact with Intermediate Filaments. Proc. Natl. Acad. Sci. USA.

[46] Jamora C., Fuchs E. (2002). Intercellular Adhesion, Signalling and the Cytoskeleton. Nat. Cell Biol..

[47] Yang J.S., Lavker R.M., Sun T.T. (1993). Upper Human Hair Follicle Contains a Subpopulation of Keratinocytes with Superior in Vitro Proliferative Potential. J. Investig. Dermatol..

[48] Cotsarelis G. (2006). Epithelial Stem Cells: A Folliculocentric View. J. Investig. Dermatol..

[49] Kobayashi K., Rochat A., Barrandon Y. (1993). Segregation of Keratinocyte Colony-Forming Cells in the Bulge of the Rat Vibrissa. Proc. Natl. Acad. Sci. USA.

[50] Han J., Chu W., Lin K., Wang X., Gao Y. (2023). β-Catenin Activation in Gli-1+ Stem Cells Leads to Reprograming of the Hair Follicle. Eur. J. Dermatol..

[51] Han J., Lin K., Choo H.Q., Chen Y., Zhang X., Xu R.H., Wang X., Wu Y. (2022). Distinct Bulge Stem Cell Populations Maintain the Pilosebaceous Unit in a β-Catenin-Dependent Manner. iScience.

[52] Gao Y., Fan Z., Xiao X., Kong D., Han J., Chu W. (2024). Epidermal ET-1 Signal Induces Activation of Resting Hair Follicles by Upregulating the PI3K/AKT Pathway in the Dermis. FASEB J..

[53] Rowden G. (1975). Ultrastructural Studies of the Keratinized Epithelia of the Mouse III. Determination of the Volumes of Nuclei and Cytoplasm of Cells in Murine Epidermis. J. Investig. Dermatol..

[54] Inoue K., Yoshimura K. (2013). Isolation and Characterization of Human Hair Follicle Epithelial Cells. Methods in Molecular Biology.

[55] Raxworthy M., Cunliffe W., Wood E. (1987). The Influence of Proteases on the Colony-Forming Efficiency of Human Keratinocytes in Culture. Biochem. Soc. Trans..

[56] Myung P., Ito M. (2012). Dissecting the Bulge in Hair Regeneration. J. Clin. Investig..

[57] Jiang S., Zhao L., Purandare B., Hantash B.M. (2010). Differential Expression of Stem Cell Markers in Human Follicular Bulge and Interfollicular Epidermal Compartments. Histochem. Cell Biol..

[58] Orazizadeh M., Hashemitabar M., Bahramzadeh S., Dehbashi F.N., Saremy S. (2015). Comparison of the Enzymatic and Explant Methods for the Culture of Keratinocytes Isolated from Human Foreskin. Biomed. Rep..

[59] Veltri A., Lang C., Lien W.H. (2018). Concise Review: Wnt Signaling Pathways in Skin Development and Epidermal Stem Cells. Stem Cells.

[60] Amoh Y., Hoffman R.M. (2017). Hair Follicle-Associated-Pluripotent (HAP) Stem Cells. Cell Cycle.

[61] Hamida O.B., Kim M.K., Sung Y.K., Kim M.K., Kwack M.H. (2024). Hair Regeneration Methods Using Cells Derived from Human Hair Follicles and Challenges to Overcome. Cells.

[62] Abaci H.E., Coffman A., Doucet Y., Chen J., Jacków J., Wang E., Guo Z., Shin J.U., Jahoda C.A., Christiano A.M. (2018). Tissue Engineering of Human Hair Follicles Using a Biomimetic Developmental Approach. Nat. Commun..

[63] Jahoda C.A.B.A.B., Horne K.A.A., Oliver R.F.F. (1984). Induction of Hair Growth by Implantation of Cultured Dermal Papilla Cells. Nature.

[64] Higgins C.A., Chen J.C., Cerise J.E., Jahoda C.A.B.B., Christiano A.M. (2013). Microenvironmental Reprogramming by Threedimensional Culture Enables Dermal Papilla Cells to Induce de Novo Human Hair-Follicle Growth. Proc. Natl. Acad. Sci. USA.

[65] Weinberg W.C., Goodman L.V., George C., Morgan D.L., Ledbetter S., Yuspa S.H., Lichti U. (1993). Reconstitution of Hair Follicle Development in Vivo: Determination of Follicle Formation, Hair Growth, and Hair Quality by Dermal Cells. J. Investig. Dermatol..

[66] Lichti U., Weinberg W.C., Goodman L., Ledbetter S., Dooley T., Morgan D., Yuspa S.H. (1993). In Vivo Regulation of Murine Hair Growth: Insights from Grafting Defined Cell Populations onto Nude Mice. J. Investig. Dermatol..

[67] Larouche D., Cuffley K., Paquet C., Germain L. (2011). Tissue-Engineered Skin Preserving the Potential of Epithelial Cells to Differentiate into Hair after Grafting. Tissue Eng. Part A.

[68] Cortez Ghio S., Le-Bel G., Lavoie A., Larouche D., Germain L., Böttcher-Haberzeth S., Biedermann T. (2019). Isolation and Culture of Human Keratinocytes. Skin Tissue Engineering: Methods and Protocols.

[69] Bisson F., Rochefort É., Lavoie A., Larouche D., Zaniolo K., Simard-Bisson C., Damour O., Auger F.A., Guérin S.L., Germain L. (2013). Irradiated Human Dermal Fibroblasts Are as Efficient as Mouse Fibroblasts as a Feeder Layer to Improve Human Epidermal Cell Culture Lifespan. Int. J. Mol. Sci..

