# Supplementary material for: Bulge-Derived Epithelial Cells Isolated from Human Hair Follicles Using Enzymatic Digestion or Explants Result in Comparable Tissue-Engineered Skin
Source: Int J Mol Sci. 2025 Feb 21;26(5):1852. doi: 10.3390/ijms26051852 (PMC11899990; doi:10.3390/ijms26051852)
Supplement: Supplementary file 1 [file ijms-26-01852-s001.zip › ijms-3426610-supplementary.pdf]

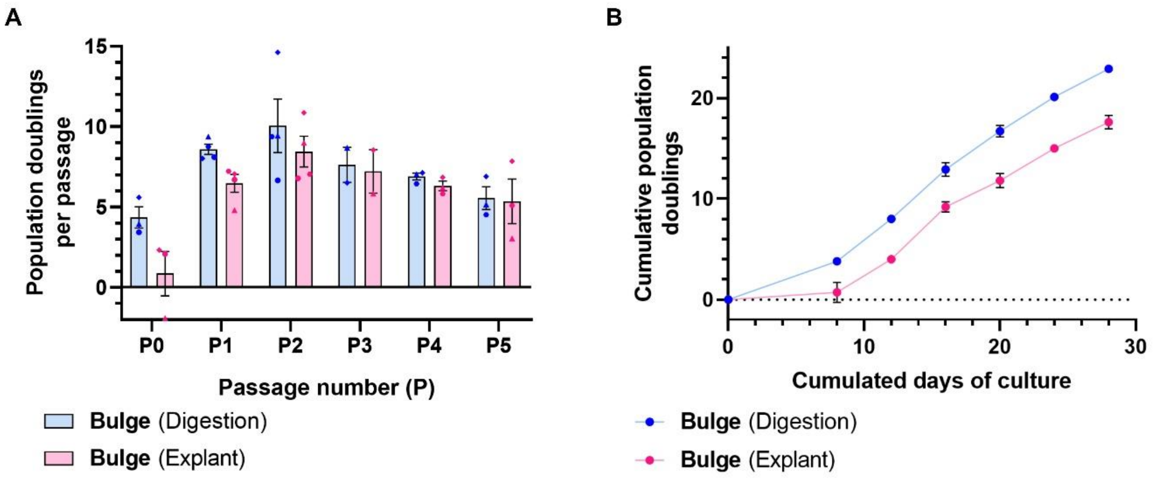

Supplementary Figure S1. Comparison of proliferation of bulge-derived epithelial cells either extracted by digestion or by explant. **A**. Population doublings per passage (8 days/passage). Data are presented as mean ± standard error of the mean (SEM). Each point represents a different donor (N). Detailed information about the donors, identified by their corresponding symbols, is provided in Table 1. **B**. Cumulative population doublings over culture time. Each point is represented as mean ± standard error of the mean (SEM).
